# Eating disorders and oral health: a scoping review

**DOI:** 10.1186/s40337-023-00778-z

**Published:** 2023-04-04

**Authors:** Rachel Presskreischer, Michael A. Prado, S. Emre Kuraner, Isabelle-Maria Arusilor, Kathleen Pike

**Affiliations:** 1 Department of Epidemiology, Columbia University Mailman School of Public Health, 722 W. 168th Street, New York, NY 10032 USA; 2 Columbia University College of Dental Medicine, New York, NY USA; 3 Fordham University, Bronx, NY USA; 4 Department of Sociomedical Sciences, Columbia University Mailman School of Public Health, New York, NY USA; 5 Department of Psychiatry, Columbia-WHO Center for Global Mental Health, Columbia University Irving Medical Center, New York, NY USA

**Keywords:** Oral health, Eating disorders, Dental education

## Abstract

**Background:**

Screening and treatment guidance for somatic sequalae of eating disorders typically include specifics such as laboratory testing, observable physical signs, and treatment interventions. Oral health guidance is notably sparse or absent from many guidelines. Often, the only mention of oral health is the potential erosion caused by self-induced vomiting and suggests a referral to an oral health professional. The guidelines generally do not include information about education and training of oral health professionals.

**Objective:**

The objective of this research was to explore the literature on eating disorders and oral health including the effects of eating disordered behaviors on oral health and training of oral health professionals to increase their capacity to recognize and appropriately address clinical care needs of individuals with eating disorders.

**Methods:**

A comprehensive scoping review was conducted to investigate what is known about the relationship between eating disorders and oral health and training provided to oral health professionals in recognition and treatment of individuals with eating disorders. The search was completed using PubMed, Embase, Science Direct, Google Scholar, and the Journal of the American Dental Association.

**Results:**

Of 178 articles returned in the initial search, 72 full texts were read, and 44 were included based on eligibility criteria. The retained articles were categorized thematically into articles related to (1) oral health professional education and training, (2) the oral health effects of eating disorders, and (3) patient experiences of oral health care.

**Conclusion:**

Most of the research on the relationship between eating disorders and oral health examines the impact of eating disordered behaviors. There is a significantly smaller literature on the knowledge and training of oral health professionals related to eating disorders and individuals with eating disorders’ experiences of oral health care. Research on education and training of oral health professionals should be expanded globally, taking into consideration the suitability of interventions for diverse models of oral health education and service delivery. Further, there is an opportunity for eating disorder professionals and professional organizations to improve understanding and care of eating disorders by building relationships with oral health providers and professional organizations in their local communities.

## Abstract (summary)

Oral health professionals, including dentists and dental hygienists, are well positioned to observe signs of eating disordered behaviors during routine oral health care. To gain an understanding of what is known about the relationship between eating disorders and oral health, we reviewed research studies that discussed any aspect of this relationship. We identified 44 studies meeting our criteria. Most of the extant research focuses on the impact of eating disorders on oral health. Smaller portions of the literature discussed education and training of oral health professionals and patients’ oral health behaviors (including routine oral health care). Educational interventions to increase oral health professionals’ knowledge of eating disorders and confidence in raising concerns with patients are effective, but evidence about whether they are being implemented in training programs is lacking. Further, many studies indicated the need to connect oral health professionals to eating disorder treatment providers. Additional research is needed to develop guidance and best practices for collaboration between fields.

## Background

Eating disorders are serious psychiatric illnesses with substantial morbidity and mortality, causing significant disturbances in somatic health and psychosocial functioning [1–3]. Research on the somatic effects of eating disorders indicates that they impact all body systems and include conditions ranging in severity from vitamin deficiencies to potentially fatal electrolyte imbalances and hypoglycemia [4]. Internationally, the findings from medical research have been translated into screening and treatment guidelines for providers by professional organizations in eating disorders, psychiatry, other medical specialties, and government bodies [5–10]. While screening and treatment guidance for many sequalae include specifics such as laboratory testing, observable physical signs, and treatment interventions, the guidance related to oral health is notably sparse or absent from many of the guidelines. Often, the only mention of oral health is the recognition that self-induced vomiting may cause dental erosion and the recommendation for eating disorder professionals (e.g., therapists, nutrition professionals, nurses, physicians who treat individuals with eating disorders) to refer someone who is vomiting to an oral health provider (OHP) such as a dentist or dental hygienist. Notably absent is guidance about when to refer patients to OHPs. This omission is particularly problematic given that eating disorder professionals report dissatisfaction with their level of oral health education, and thus tend to wait until a patient reports complications [11]. Guidance for OHPs on the recognition and clinical care of someone with an eating disorder is even more sparse.

Across health conditions, it is widely understood that early identification and intervention leads to better health outcomes. The same is true for people with eating disorders. The paucity of guidance regarding the link between oral health and eating disorders means that health conditions are not addressed until the impact is severe. A significant benefit of early intervention is that it decreases the time of untreated illness [12], which is associated with positive outcomes such as shorter time to remission [13]. Despite this opportunity, medical education regarding eating disorders is limited. In a study of 637 U.S. medical education residency programs in internal medicine, pediatrics, family medicine, psychiatry, and child and adolescent psychiatry, 514 did not offer any rotations for eating disorders. In the 123 programs with rotations, only 42 had a formal, scheduled rotation [14]. A study of Canadian medical residents found that participants had, at most, 5 h of training on eating disorders; those who had such training reported comfort with screening for and assessing eating disorders, but a lack of comfort with medical management. An evaluation of the residents’ knowledge of assessment and treatment of eating disorders supported that they had sufficient knowledge of assessment practices but were not well-versed in management and treatment [15]. Medical education in the United Kingdom is similarly lacking in education about eating disorders. A 2018 study noted that most physicians receiving < 2 h of instruction across 10–16 years of training. Additionally, eating disorders are absent from the curricula of 20% of medical schools, and < 1% of students have access to specialty clinical experiences [16].

Given the particular effects of eating disorders on oral health, and the opportunity for oral health providers to play an instrumental role in early detection of eating disorders, the purpose of this study was to assess the status of research on clinical implications and provider education about eating disorders for oral health providers. Additionally, as the reported rates of eating disorders education and training are generally low, the question remains whether such training is provided for OHPs given the high rates of oral health symptoms that individuals with eating disorders experience. Presently, there is limited research on oral health and eating disorders, with existing systematic reviews focusing on clinical presentation. The primary aim of this study is to examine the literature on eating disorders and oral health broadly to capture clinical research (e.g., effects of eating disordered behaviors on oral health and treatment strategies) and topics such as dental education and training.

## Methods

A scoping review was conducted to identify key concepts, sources of evidence and research gaps at the intersection of eating disorders and oral health. Scoping reviews are appropriate in cases where there is uncertainty about the breadth of the literature on a topic. Accordingly, the study was conducted with the methodology and guidance in Peters et al. and from PRISMA [17, 18]. A search was conducted in Pubmed, Embase, Google Scholar, the Journal of the American Dental Association and Science Direct. Search terms included “Eating Disorder”; “Eating Disorders”; “Disordered Eating”; “ED”; “EDs”; “ED's”; “Anorexia”; “AN”; “Bulimia”; “BN”; “EDNOS”; “Binge Eating Disorder”; “Restrictive Food Intake Disorder”; “ARFID”; “Rumination Disorder”; “Other Specified Feeding or Eating Disorder”; “OSFED”; “Unspecified Feeding or Eating Disorder”; and “UFED”. These terms were combined with the additional terms “Dentist”; “Dentists”; “Dental”; “Dentistry”; “Oral Hygiene”; “Hygienist”; “Hygienists”; “Oral Health”; “Caries”; “Cavity”; “Cavities; Teeth”. MeSH and Emtree terms included “Feeding and Eating Disorders”[Mesh]) AND “Oral Health”[Mesh] Emtree terms: ‘dentistry’/exp/mj AND ‘eating disorder’/exp/mj. Articles were eligible for inclusion if they were written in or translated into English, published in peer-reviewed journals, and had a publication date after the year 2000. The year 2000 was selected for three reasons. First, to capture research from the current century. Second, knowledge of eating disorders had spread beyond the mental health fields such that it would be feasible to capture research in a broader range of disciplines. Finally, the expansion in internet use at the turn of the century and growth technology would ensure that we were identifying current trends in education and training. Full inclusion and exclusion criteria are provided in Table 1. Two members of the study team reviewed all studies independently and any disagreements were discussed to reach consensus. For each article meeting inclusion criteria, data were extracted for: (1) country in which data were collected, (2) primary study aim, (3) study design, (4) included eating disorder diagnoses, and (5) findings.

**Table 1:** Inclusion and Exclusion Criteria

	Inclusion criteria	Exclusion criteria
Year of publication	1/1/2000—Present	Prior to 1/1/2000
Language	English, or English translation	Non-English, no English translation
Article type	Original research published in peer-reviewed journals	Articles not peer-reviewed, prior systematic reviews/evidence reviews, commentaries, dissertations, white papers
Study population	Individuals of any age and gender with a diagnosed with or at risk for DSM IV or DSM 5 eating disorder	Individuals without eating disorders
Focus of article	Topics relating to oral health/oral healthcare and eating disorders	Oral health topics unrelated to eating disorders, eating disorder related topics not relevant to oral health

## Results

**Fig. 1:**
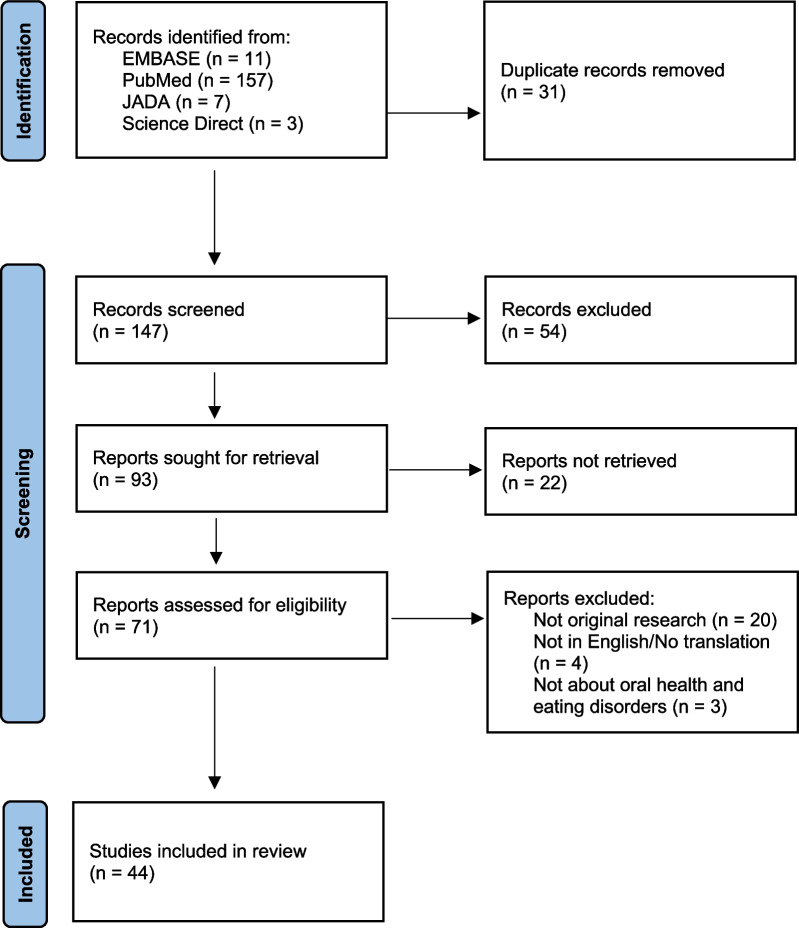
PRISMA flow diagram of study selection. *From:* Page MJ, McKenzie JE, Bossuyt PM, Boutron I, Hoffmann TC, Mulrow CD, et al. The PRISMA 2020 statement: an updated guideline for reporting systematic reviews. BMJ 2021;372:n71. https://doi.org/10.1136/bmj.n71

An initial 178 articles were identified from the search terms (Fig. 1). After removal of duplicates, 147 titles and abstracts were screened for inclusion. A total of 93 articles were sought for retrieval, with 21 not retrieved due to a lack of full-text availability. Of the remaining 72 articles, 20 were excluded as not original research, 4 lacked an English language version, and 3 were excluded because they were not relevant to oral health and eating disorders. Forty-four studies were retained for inclusion (Table 2). Of these 44 studies, seventeen related specifically to oral health professionals [19–35], twenty addressed the impact of eating disorders on oral health [36–55], and seven focused on patient experiences and perspectives [56–62].

**Table 2:** Articles included in the scoping review

Author (Year) Country	Study aim(s)	Study design	Included ED diagnoses	Findings
Back-Brito [36] Brazil	To evaluate fungal microflora in the oral cavity	Matched case–control N = 59	AN, BN	The identification of uncultured microflora species first reported in this study was also observed in dental biofilm from the ED group
Boillot [37] France	To determine whether generalized reduced periodontium and dental erosion in women with EDs are associated with systemic biological parameters	Secondary analysis of the PERIOED case–control study N = 45 Female	AN, BN	Serum ferritin levels together with age may be helpful in screening patients with ED for adequate referral to a dentist
Burgard [19] USA	To better understand how dental practitioners identify, counsel, and refer patients with EDs	Survey N = 123	N/A	Approximately 50% of respondents reported evaluating a patient with an ED in the past year and most spoke to the patient or a parent. Some respondents do not raise concerns due to ambiguity of dental findings or not knowing what to say. Less than 1/3 referred patients to additional care, and those who did, referred almost exclusively to primary care
Conviser [56] USA	To understand oral health behaviors after purging and patient perspectives on barriers to patient-initiated discussion of EDs with OHPs	Cross-sectional survey N = 201	BN	Participants had high levels of concern about their oral health and a high incidence of oral health problems, but less than one third considered their OHPs to be the most helpful source of oral health information
De Moor [38] Belgium	To present a case of a female patient with a history of AN, followed by BN that resulted in dental destruction over a 5-year period	Case study N = 1	AN, BN	Early erosions were particularly limited to the lingual surfaces of the maxillary anterior teeth and occlusal surfaces of the maxillary premolars and mandibular posterior teeth. Resin restorations have been used to restore teeth with erosion and those demonstrating dentine hypersensitivity
DeBate [27] USA	To determine the knowledge among dentists and dental hygienists concerning the oral and physical manifestations of EDs	Randomized cross-sectional study N = 945	N/A	Most respondents had low knowledge of clinical findings associated with EDs, with hygienists more likely to identify oral manifestations. There was generally low knowledge of physical manifestations of EDs
DeBate [26] USA	To explore beliefs, attitudes, and experiences of general dentists regarding ED-specific secondary prevention behaviors	90-min focus groups N = 21	N/A	Curriculum development, policies, and practices are places for intervention to support and sustain secondary preventive clinical behaviors among dentists
DeBate [22] USA	To explore readiness and capacity for integration of oral health and mental health services	Cross-sectional survey N = 378	N/A	Only 18% of the sample reported referring patients to clinical eating disorder care. It is important to improve capacity for secondary prevention and self-efficacy
DeBate [28] USA	To assess sex differences among dentists pertaining to current behaviors and behavioral beliefs about EDs	Cross-sectional survey N = 350	N/A	Respondents had an overall low level of secondary ED prevention practices. Dissonance was observed between dentists about reported high levels of perceived benefits of prevention practices and self-efficacy and the modest number of dentists currently engaged in secondary prevention practices. More female dentists reported engagement in prevention practices than their male counterparts
DeBate [25] USA	To assess the breadth and depth of ED specific comprehensive and primary care instruction in dental and dental hygiene curricula	Cross-sectional survey N = 114	N/A	The findings indicate an increased inclusion of ED training in programs from a previous study. More dental hygiene programs include ED education than dental programs. There is a significant lack of training in communication with patients exhibiting signs of EDs
DeBate [23] USA	To develop and evaluate a program to improve secondary prevention of EDs in oral health providers	Two phase study: Phase 1: focus groups, N = 41 Phase 2: pilot evaluation, N = 64	N/A	There was a statistically significant change from pre intervention to post intervention across variables. Participants found training useful, provided more information than generally available, and was appropriately tailored
DeBate [21] USA	To develop and evaluate a framework aimed at oral healthcare providers to engage in active secondary prevention of EDs	Group randomized controlled trial N = 18	N/A	There was significant improvement from pre-intervention to post-intervention in the intervention group compared to the control group on knowledge of EDs and oral findings, skills-based knowledge and self-efficacy
DeBate [24] USA	To test whether an interactive, web-based training program is more effective than an existing, latest e-learning program at improving oral health students’ knowledge, motivation, and self-efficacy to address signs of disordered eating behaviors with patients	Group randomized controlled trial N = 18 classes, 317 students	N/A	Post-test differences between groups showed greater improvement among intervention participants in three domains compared to the alternative program. The findings suggest that interactive training programs may be better than e-learning
DeBate [20] USA	To use IM to develop a theory- and evidence- based intervention to increase the capacity of oral health providers to engage in secondary prevention of EDs	Prospective group-randomized controlled trial N = 27	N/A	The systematic IM process resulted in an innovative theory and evidence-based training curriculum that highlighted the importance of oral-systemic health and associated clinical behaviors that can positively impact the overall health of persons with ED behaviors
DiGioacchino [29] USA	To build a framework for the development of effective programming to increase the proportion of dental practitioners involved in secondary and tertiary prevention and case management	Pen and paper survey N = 45	N/A	The findings indicate that dentists and hygienists were not aware of complications/manifestations of EDs. There was a lack motivation to take action, with identified barriers to action of difficulty speaking to patients about concerns of ED and lack self-efficacy in corresponding with primary care providers or referral processes
Dynesen [39] Denmark	To study if BN has an impact on salivary gland function, if salivary gland function relates to dental erosion in persons with BN, and if dental erosion in BN is related to behavior regarding diet, purging, and duration of the ED	Case–control, questionnaire and dental exam N = 40	BN	The BN persons had impaired unstimulated whole saliva (UWS), mainly owing to medication; increased feeling of oral dryness; and more dental erosion. Dental erosion was related to the duration of ED, whereas no effect of vomiting frequency or intake of acidic drinks on reduced UWS was observed
Dynesen [57] Denmark	To uncover knowledge, experience, and attitudes of oral health and oral health behavior among persons with EDs	Cross-sectional, electronic questionnaire N = 260	AN, BN, atypical AN	The participants with EDs were, in general, concerned about their teeth. Some feared that they had severe and irreversible tooth damage, and many were overly occupied with oral hygiene procedures. Some of the participants had good experiences in communicating with the dentist and wanted the dentist to address EDs in the clinic. However, participants with less-positive experiences highlighted a need for dentists with specialized knowledge of EDs and communication skills, focusing on an empathic approach from the dentist
Emodi-Perlman [40] Israel	To compare the prevalence of psychologic, dental, and temporomandibular disorder signs and symptoms between young women suffering from chronic EDs and a control group of age-matched, healthy women, and to evaluate the impact of frequent vomiting on these signs and symptoms among the ED group	Clinical examination and self-administered questionnaires N = 129	AN, BN, EDNOS	Women with EDs presented higher general muscle sensitivity than healthy women of the same age, as well as higher emotional and psychologic distress. This may suggest a higher susceptibility of women suffering from ED to suffer also from myofascial pain than healthy subjects. Most differences did not reach statistical significance, probably because of the small number in each group and the application of the Bonferroni correction for multiple testing. However, the results clearly indicate some disparity between the 2 groups
Frimenko [30] USA	Assess dental students' ED and interprofessional care (IPC) related educational experiences, perceptions of preparedness for ED-related communication with patients and providers, and attitudes related to an IPC approach to EDs	Paper and web-based survey N = 596	N/A	3rd and 4th year students felt educated about basic ED related issues (signs, symptoms, systemic effects) but not about ED treatment protocol and referral protocol. Reported asking about physical health and referring but not mental health and rarely referred to mental health provider. Did not feel prepared to communicate with patients and health care providers from other disciplines about mental health
Galindo [31] USA	To describe the reconstruction of a maxillary anterior segment using immediate implant placement and immediate implant loading techniques, aided by computer- guided implant treatment software and stereolithographic models and surgical templates, in a patient with a history of ED	Case study N = 1	BN	This clinical report describes rehabilitation using implant- supported FPDs on immediately placed and immediately loaded dental implants, aided by computer-guided implant treatment software, stereolithographic models, and surgical templates, of a partially dentate patient affected by EDs
Giraudeau [32] France	To assess the benefits and feasibility of providing a systematic dental examination via telemedicine for all patients in the ED day hospital	Two phase study: virtual visit and analysis N = 50	AN, BN	Dental erosion was found in 92% of the patients, and 50% had at least one tooth with a BEWE score of 2 or 3
Hermont [41] Brazil	To compare the occurrence of tooth erosion and dental caries in adolescents with and without risk behavior for EDs	Controlled cross-sectional study N = 1,203	N/A	Severe risk behavior for EDs was significantly associated with tooth erosion, but not with dental caries
Imai [42] Japan	To present a case of necrotizing sialometaplasia (NS) accompanied by significant dental erosion of the maxillary teeth of the palatal surfaces owing to chronic self-induced vomiting	Case study N = 1	N/A	Temporary worsening of binging-purging before the development of NS would likely have led to an increase in traumatic stress and chemical exposure of the palate. Iron deficiency hypochromic anemia could compromise the steady supply of oxygen to the palate locally affected by frequent abnormal eating behaviors. These systemic and local factors might trigger irreversible ischemic changes in the palatal salivary gland tissues for a short time
Johansson [43] Sweden	To compare the oral health status of patients with EDs, with sex- and age-matched controls, with a view to identify self-reported and clinical parameters that might alert the dental healthcare professional to the possibility of EDs	Matched case–control, questionnaire and dental exam N = 108	AN, BN, EDNOS	Self-reported presence of dental problems was significantly more common among ED patients compared with controls, and the presence of oral problems on a daily basis was reported by 13% of ED patients compared with only 2% of the controls. The reporting of symptoms on a weekly or daily basis was about two- to three-fold higher in ED patients than in controls
Johansson [33] Norway	To investigate knowledge, attitudes and clinical experience about patients with EDs among Norwegian dentists	Questionnaire sent via mail N = 1726	N/A	The dentists in this study reported limited clinical experience and insufficient knowledge regarding EDs. There is therefore a need to increase both undergraduate and continuing education in this field
Johansson [59] Sweden	To investigate diet, oral hygiene habits and awareness of possible negative factors for oral health, as well as utilization of dental care in ED patients during periods when self-perceived ED status was "relatively good" vs. "relatively bad”	Case–control N = 108	AN, BN, EDNOS	The conclusions drawn from this study are that ED patients presents a number of dietary and other types of behavior that are potentially harmful for their general and oral health. For a more accurate detection of these activities, it is important that the patient report on the behaviors both when she/he is in a relatively good as well as being in a more active disease state
Johansson [58] Sweden	To investigate the behavior in ED patients with self-induced vomiting in relation to binge eating, oral health, and dental care	Cross-sectional questionnaire N = 17	AN, BN, EDNOS	Variation in frequency and duration of episodes of self-induced vomiting indicate that the consequences for oral health will vary and be affected by the specific compensatory behavior executed in patients suffering from an ED. The dental team should be made aware of the likely detrimental effects of binge eating and vomiting on oral health and the large variations of behavior and the cyclical nature of the disease
Lee [34] Korea	To present case studies that describe the prolonged food restriction of two girls due to the wearing of dental braces in which pathological eating behaviors and serious medical conditions emerged	Case study N = 2	ARFID, AN	Pathological eating problems may occur in association with orthodontic treatment during adolescence. To prevent serious eating problems that are related to fatal physical conditions, a collaborative assessment between the orthodontist, the patient, the family, and a psychiatrist about the earliest signs of EDs is required
Lifante-Oliva [44] Spain	To study oral complications in females with EDs	Descriptive study N = 17	ED diagnoses in DSM IV	A significant alteration in oral tissue occurs; this has an adverse impact on oral health, producing an accumulation of local irritants which favor the appearance of oral diseases
Lourenço [45] Portugal	To evaluate the oral health status and orofacial problems in a group of outpatients with EDs	Case–control, questionnaire and dental exam N = 33	AN, BN	Outpatients with EDs were found to present a higher incidence of oral-related complications and an inferior oral health status, compared to gender- and age-matched controls
Lundgren [46] Denmark	To determine if nocturnal eating is related to tooth loss in a large, epidemiologic sample	Longitudinal, case–control design N = 2217	NES	The study findings show that nocturnal eating does have oral health implications, supporting previous findings that nocturnal eating, but not evening hyperphagia, is associated with tooth loss, periodontal disease and active tooth decay
Otsu [47] Japan	To investigate the relationship between the severity of dental erosion and the vomiting behavior and regular dietary habits of patients with EDs	Oral assessment for dental erosion, standardized interview N = 71	AN, BN, EDNOS	While self-induced vomiting is the main cause of dental erosion in ED patients, the erosion severity may be affected by behavior when inducing vomiting or by routine consumption of certain foods and beverages. Addressing these factors may help prevent severe dental erosion in patients who chronically induce vomiting
Pallier [48] France	To evaluate dental and periodontal health in anorexia nervosa and bulimia nervosa patients	Case–control full-mouth exam and review of oral hygiene behaviors N = 140	AN, BN, EDNOS	The study found a significantly different oral disease profile among patients as a function of ED diagnosis type. AN patients presented worse periodontal conditions with higher dental plaque accumulation, gingival inflammation and clinical attachment loss than BN patients. AN patients also reported brushing their teeth more frequently than BN patients (p < 0.01). The findings suggest an adaptation to the prevention of oral diseases according to the ED diagnosis type
Paszynska [50] Poland	To evaluate stimulated and resting salivary flow rate and the activity of the following enzymes in both types of saliva: amylase, aspartate amino transferase (AST), alanine amino transferase (ALT), collagenase, lysozyme, peroxidase, serine and acidic proteases, and trypsin in persons with AN and to compare them with those of healthy controls	Case–control observational N = 66	AN	Reduced salivary flow might be one indicator of anorexia. Despite starvation and anorexia development, salivary key enzymes show physiological activity. This indicates a partial adaptation of the organism to severe conditions during malnutrition
Paszynska [49] Poland	To establish the oral status regarding caries incidence, tooth wear, gingival inflammation, and oral hygiene levels among severely ill adolescent inpatients diagnosed with AN	Case–control cross‐sectional survey, clinical examination of dental wear N = 220	AN	Study findings indicate impaired dental and gingival conditions in young people with anorexia. Considering AN’s potential role in oral health, it is essential to monitor dental treatment needs and oral hygiene levels in their present status to prevent forward complications in the future
Rangé [51] France	To design a questionnaire to identify risk factors and symptoms of oral diseases and to test its reliability as a self-report form among people with AN	Self-report questionnaire, face-to-face repeat questionnaire administered by dentist N = 69	AN	The study authors developed and tested a questionnaire that identifies risk factors and symptoms of oral diseases in anorexia nervosa. The 26-item form of the questionnaire (long version) is moderately reliable as a self-reported form. A short version of the questionnaire, including the 10 most reliable items, is recommended for oral risk assessment in patients with anorexia nervosa. The clinical value of the self-administered questionnaire remains to be evaluated
Sales-Peres [52] Brazil	To evaluate the prevalence, distribution, and associated factors of dental wear among patients with EDs	Case–control N = 60	AN, BN	Dental wear was similar for both groups; the experimental group presented more moderate wear in molars. The etiological factors of tooth wear related with dental wear were biting objects and pain in temporomandibular joints
Sharifian [60] Finland	To evaluate the associations between dental fear and EDs and BMI, with respect to age, gender, educational, sector, attitude to food, and mental well-being among a representative sample of Finnish university students	Cross-sectional secondary analysis of data from University Student Health Survey N = 3090	No diagnosis, AN, BN, Other	Among the Finnish university students BMI in males and problems of mental well-being in females were positively associated with high dental fear. The results of this study support possible common vulnerability factors that dental fear and other psychological disorders may share
Shaughnessy [53] USA	To evaluate the dental and periodontal health of adolescents and young women with restrictive anorexia nervosa, and the relationship between bone mineral density assessed by dual energy X-ray absorptiometry (DXA) and dental radiographs	DXA bone mineral density measurements, comprehensive dental examination N = 23	AN	Despite subnormal DXA measurements in most patients, essentially all adolescents had a normal dental examination. Dental providers should be cognizant of the fact that many patients with EDs may not display the “classic” findings reported in the literature
Sirin [61] Turkey	To evaluate the levels of dental fear and anxiety in women with EDs scheduled for oral surgery	Case–Control N = 562	AN, BN, EDNOS	The findings indicate that the ED group had greater levels of dental anxiety and fear than the non-ED dental patients and randomly selected individuals from a nonclinical environment. In addition, significant differences were found between the AN subtypes and the presence or absence of purging behavior
Strużycka Poland [55]	To investigate the prevalence of erosive lesions and related risk factors in the population of 18-year-old young adults in Poland	Cross-sectional clinical assessment of dentition, questionnaire N = 1869	N/A	In this study, 1.4% reported EDs and prevalence of erosion in anterior region significantly associated with EDs. Roughly half of those who had EDs had varying intensity of erosion type damage to the teeth
Turhani [35] Austria	To address complications and complex management of an active bulimic patient treated with dental implants and elucidate partial failures resulting in mandibular fracture	Case study N = 1	BN	Dental implant in osteopenic bone predisposes atrophic mandibles for infection and subsequent fracture. Risk/benefit ratio of endosseous implants is not favorable long term, thus people with EDs need to be diligently selected and fully informed of the hazards underlying this type of rehab
Willumsen [62] Norway	To investigate the prevalence of dental fear, dental attendance, reports of dental erosion, and frequency of informing dentists about eating disorders in women with EDs	Questionnaire via mail N = 371	AN, BN, BED	The survey supports the hypothesis that women with EDs have higher levels of dental fear than women in the general population. The present study further indicates that dental fear is a risk factor for poor dental health in ED patients
Ximenes [54] Brazil	To examine the prevalence of oral alterations related to EDs and associated factors	Cross-sectional self-report questionnaires, dental examination N = 650	N/A	Significant associations were observed in mucositis, cheilitis, hypertrophy of salivary glands, and dental erosions. The prevalence of adolescents at risk for EDs was of 33.1%, according to EAT-26 and 1.7% (high scores) and 36.5% (medium scores) in BITE. All these factors showed significant relation to EDs

*ED* Eating disorder, *AN* Anorexia nervosa, *BN* Bulimia nervosa, *BED* Binge eating disorder, *EDNOS* Eating disorder not otherwise specified which included BED (replaced with other specified feeding or eating disorder and unspecified feeding or eating disorder in DSM-5), *NES* Night eating syndrome, *ARFID* Avoidant/restrictive food intake disorder, *DSM-IV* The Diagnostic and Statistical Manual of Mental Disorders—4th edition

### Oral health professional education and training

The 17 articles about oral health professionals fell into three overarching categories: knowledge, education, and practices.

Seven of the articles assessed OHPs’ knowledge of the oral health signs of eating disorders, symptoms of eating disorders, how to raise concerns with a patient, and where to refer a patient for treatment if necessary. Of these seven articles three studies included dentists and dental hygienists [19, 27], three included dentists only [26, 28], one included hygienists only [22], and one included a random sample of dental practices. All studies were conducted in the United States except one which was conducted in Norway [33]. All seven studies indicated that OHPs lacked sufficient knowledge of eating disorders and limited clinical experience. Of those who were aware of oral health signs and symptoms, many reported not knowing how to address their observations with patients. Most OHPs did not refer patients for evaluation and treatment of their eating disorder, and when they did it was usually to primary care physicians rather than mental health providers.

Additional studies addressed education provided to oral health students about eating disorders and interventions to improve student and provider knowledge. The study populations included deans of dental schools and directors of dental hygiene programs [25], dentists, hygienists and students in dental and dental hygiene programs [23], and dental and dental hygiene students [20, 21, 24, 30]. The primary findings from these studies indicated that dental and dental hygiene students receive minimal instruction (between 17 and 35 min) in eating disorders [20, 25] and that interventions to provide education about eating disorders and their related effects on oral health can improve OHPs knowledge and capacity to intervene in patient care. DeBate et al. [20] study leveraged intervention mapping to design and test an online intervention to improve OHP student’s awareness of the impact of eating disordered behaviors on oral health and treatment options available to people with eating disorders [20]. The study demonstrated positive benefit, high student satisfaction with the program, and student interest in retaining access to the educational materials provided in the intervention.

The final category of articles relates to specific practices of OHPs when working with patients who have eating disorders. Of the four articles in this category, three are case studies providing insight into procedures OHPs used to address specific effects of eating disorders on oral health. In a 2014 paper, Lee et al. present two cases of adolescents who restricted food intake as a result of wearing braces that led to hospitalization for eating disorders [34]. The two other case studies describe procedures for treating oral health conditions in patients with longstanding bulimia nervosa [31, 35]. In both cases the eating disorder had significant effects on the patient’s teeth, including placement of implants or removable prosthesis, addressing both functional and aesthetic concerns. A final study examined the integration of tele-dentistry consultations into eating disorder day treatment to screen for oral health conditions and prevent dental erosion [32]. The article findings suggest that the use of tele-dentistry in an established eating disorder treatment program may provide advantages such as targeted evaluation of the oral health of individuals with eating disorders and an opportunity to establish oral health care for patients who may otherwise not have access to or not seek care.

### Effects of eating disorders on oral health

The largest numbers of articles generated by the scoping review focused on the oral health effects of eating disorder behaviors. One study examined the prevalence of erosive lesions in a cross-section of Polish 18-year-olds and found that lesions were significantly associated with eating disorders [55]. Eleven of the studies used case–control designs to compare oral health findings between people with/at risk for eating disorders and healthy controls/those not at risk. An additional six studies described oral health effects and risk behaviors in patients with diagnosed eating disorders [41, 44, 47, 51, 53, 54]. Findings from these studies indicate negative effects of eating disorders on oral health including tooth erosion, increased size of salivary glands, and gingival recessions at higher rates than control groups. One study evaluated dental conditions and oral health behaviors (e.g., frequency of brushing) and noted differences in the presentation of both between people with anorexia nervosa and bulimia nervosa [48]. Additional findings in these studies showed increased self-reported oral health problems in individuals with eating disorders compared to those without [43], and a higher presentation of general muscle sensitivity potentially suggesting higher susceptibility to myofascial pain than healthy subjects [40].

Two articles described the impact of eating disorder behaviors in individual cases. One study described a patient who’s self-induced vomiting led to necrotizing sialometaplasia (a benign ulcerative lesion due to tissue death of the salivary glands) and significant dental erosion [42]. The other case study described dental evaluation and treatment of a patient over a 6-year period, noting worsening oral health symptoms and denial of an eating disorder [38]. At the appointment 6 years from the original appointment, she shared her longstanding eating disorder with her OHP and her initial reluctance to confirm their concerns.

A final study designed and tested a questionnaire to identify oral health risk factors and symptoms in individuals with anorexia nervosa. A 26-item questionnaire was assessed and found have moderate reliability when administered as a self-report form. The ten most reliable items from the original questionnaire were recommended as a risk assessment in patients with anorexia nervosa [51].

### Patient practices and experience of oral health care

The remaining seven articles evaluated dental fear and anxiety, oral hygiene knowledge and attitudes, and oral health behaviors in individuals with or at-risk-for eating disorders. Three articles identified elevated dental fear and anxiety among individuals with or at risk for eating disorders compared to those without [60–62]. These study samples were Finnish university students, Norwegian women with diagnosed eating disorders recruited from a self-help organization, and Turkish individuals with and without eating disorders about to undergo oral surgery. The methods used to identify patients with or at risk for an eating disorder varied across studies, as did their assessments to measure dental fear and anxiety.

Two papers evaluated oral health concerns, sources of information about oral health effects of eating disorders, and willingness to see OHPs in people with eating disorders [56, 57]. Both articles found that participants were concerned (with high proportions expressing significant concern) about the impact of their eating disorder on their oral health. For sources of information, Conviser et al. found that, of participants who sought information about how to minimize damage from purging, 84% found the internet to be one of the most helpful sources of information, whereas only 29% included OHPs as one of the most helpful. In Dynesen et al. research, 70% obtained information about oral health complications from media sources (e.g., internet, television) compared to 24% from a dentist.

Conviser et al. and Dynesen et al. also evaluated oral care behaviors following self-induced vomiting. Conviser et al. asked whether participants rinsed the mouth with water or a mouth rinse (84%) or brushed their teeth immediately after (33%) purging. Dyneson et al. study summarized the findings of participants’ oral health behaviors following SIV as neutralizing acid in the mouth (34%) and avoiding toothbrushing (29%). Both studies, along with Wilumsen et al. [62], and Johanssen et al. [58] found that most participants had not told an OHP about their eating disorder. Johanssen et al. reported that only 6% of participants disclosed their eating disorder, with 29%, 32%, and 39% disclosing in Conviser, Dynesen, and Willumsen respectively.

Additionally, Dynesen, Willumsen, and Johanssen asked participants about frequency of visits to an OHP. Dynesen et al. framed the question in terms of frequency, with 33% reporting visiting the dentist more than twice per year [57]. Willumsen et al. asked about the date of participants’ last dental treatment—with 87% having seen a dentist in the last 2 years [62]. Johanssen et al. found that 71% attended “regular dental visits” but did not specify a time frame. However, the average time between visits for people attending any dental appointments in the study was 14 months [58].

The last study examined differences in behaviors in individuals with eating disorders at different clinical presentations in their eating disorders. They compared participant responses when symptoms were relatively absent (defined as ED-good) and when they were more “active” or highly symptomatic (defined as ED-bad) [59]. They found that behaviors associated with different states of an individual’s eating disorder posed differential risks to oral health. Compared to health controls, ED-good was predicted by the variables: higher intake of caffeinated beverages, and lower intake of regular (non-diet) soft drinks. Predictive variables of ED-bad were: lower frequency of lunch, and lower intake of sweet biscuits. A key takeaway from the study is that between ED-good and ED-bad states, an individual’s behaviors pose different risks to oral health.

## Discussion

This scoping review investigated the state of evidence about oral health and eating disorders. In addition to literature on the oral health sequalae of eating disorders, included articles addressed (1) eating disorder knowledge and education of OHPs, (2) interventions OHPs use to treat effects of eating disorders, and (3) patient attitudes and behaviors related to oral health care. Across the different categories of studies, a consistent finding was that OHPs do not receive sufficient education and training to address eating disorders in practice—inhibiting early identification, treatment, and referral.

OHPs’ insufficient knowledge of eating disorders is evidenced by an absence of or minimal educational content on eating disorders and lack of clinical exposure to patients with eating disorders in training programs. The insufficient training received in OHP training programs is similar to medical training programs [14–16, 63]. The factors that impacted OHP’s ability to identify and comfort with treating patient with eating disorders was associated with their exposure to clinical cases during training and working with a clinician who had a particular interest in eating disorders, factors also associated with physicians’ ability to identify and treat patients with eating disorders [63].

Research examining methods to educate OHPs about eating disorders found promising results. Knowledge about eating disorders and their presentation was increased, and the interventions were considered acceptable to participants. While both a static e-learning training and an interactive training improved participants’ knowledge of eating disorders, the interactive training was superior at reducing OHPs’ perceived barriers to secondary prevention, increasing perceived benefits of secondary prevention, and increasing perceived self-efficacy to perform secondary prevention behaviors [24]. The small body of research on educational programs warrants further study to examine the transferability of these interventions into non-U.S. oral health training programs. Additionally, uptake of these educational programs by dental and dental hygiene programs will be an important are for additional research.

One major area of note across the included studies was the assertion that OHPs need to be connected to eating disorder professionals. No studies were identified that examined mechanisms for connecting OHPs and eating disorder professionals. The eating disorders field has an opportunity to build relationships with local and national oral health provider organizations and with local providers to increase awareness of referral resources and offer support to OHPs who may be among the first providers to observe an eating disorder. Increasing OHPs awareness of resources for eating disorder treatment and eating disorder treatment providers’ knowledge of oral health risks and referral resources presents an opportunity for research into methods to incorporate OHPs in eating disorder treatment teams. The clearly stated need for these relationships and the noted lack of research indicates an important area of study for researchers across the globe.

A small portion of the research in this review examined eating disorder patients’ oral hygiene behaviors and feelings about oral health procedures. The findings in these studies indicate that many individuals do not disclose their eating disorder to OHPs and may engage with oral health care less frequently than clinically recommended. Future research should evaluate whether and how eating disorder treatment professionals can contribute to patient engagement with oral health care. In addition to the potential for engagement between eating disorder professionals and OHPs, there is an opportunity for eating disorder professionals to encourage patients to disclose to OHPs and to address fear and anxiety about oral health procedures. There is also an opportunity to examine the ways that OHPs can be formally included in the American Psychiatric Association practice guidelines for the treatment of eating disorders as members of the multi-disciplinary research team—and whether doing so increases engagement with OHPs.

A final note on the findings of this scoping review is that the majority of work on oral health and eating disorders is on the oral health sequalae of eating disordered behaviors. This body of literature has been the subject of previous systematic reviews [64–67]. One key issue raised in many of the studies is that the oral health effects of eating disorders are not unique to eating disordered behaviors. Most of the associated conditions (e.g., tooth erosion, susceptibility to caries, changes in salivary flow, periodontal disease) can be indicative of many other conditions. This finding, paired with the research indicating OHPs’ lack of confidence in their ability to communicate about eating disorders, suggests a need for additional research. Research on interventions to increase OHP confidence in their ability to raise concerns about disordered eating behaviors will be vital to promoting secondary prevention efforts. Additionally, research is needed on whether increasing OHPs’ communication capacity impacts patients’ willingness and comfort with disclosure of eating disordered behaviors with their providers.

This research has a number of limitations. First, articles were only included if they were available in English. Several studies returned in the search were excluded based on language that may address some of the topics that were less well represented in this study. Additionally, we did not evaluate the quality of the research. The quality of evidence for oral health sequalae of eating disorders has been previously reviewed. Future research should consider the quality of studies evaluating provider education and training and patient experiences.

## Conclusion

This scoping review sought to assess the state of research on eating disorders and oral health. While there has been significant research on the impact of eating disorders on oral health, there is a need for research in all other aspects of the intersection between eating disorders and oral health. In addition, there is a clear need to establish relationships between oral health professionals and eating disorder treatment professionals. These relationships would improve patients’ referral to specialty care when symptoms are observed in an oral health setting and increase the potential for improved oral hygiene and clinical outcomes for individuals with eating disorders.

## Data Availability

Not applicable.

## Abbreviation

OHP: Oral health professionals

## References

[1] Halmi KA, Agras WS, Robinson A (2018). Psychological comorbidities of eating disorders. The oxford handbook of eating disorders.

[2] Mehler PS, Agras WS, Robinson A (2018). Medical complications of anorexia nervosa and bulimia nervosa. The oxford handbook of eating disorders.

[3] Mitchell JE (2016). Medical comorbidity and medical complications associated with binge-eating disorder. Int J Eat Disord.

[4] Voderholzer U, Haas V, Correll CU, Korner T (2020). Medical management of eating disorders: an update. Curr Opin Psychiatry.

[5] Couturier J, Isserlin L, Norris M, Spettigue W, Brouwers M, Kimber M (2020). Canadian practice guidelines for the treatment of children and adolescents with eating disorders. J Eat Disord.

[6] Hay P, Chinn D, Forbes D, Madden S, Newton R, Sugenor L (2014). Royal Australian and New Zealand college of psychiatrists clinical practice guidelines for the treatment of eating disorders. Aust N Z J Psychiatry.

[7] Hornberger LL, Lane MA, Committee on A (2021). Identification and management of eating disorders in children and adolescents. Pediatrics.

[8] Klein DA, Sylvester JE, Schvey NA (2021). Eating disorders in primary care: diagnosis and management. Am Fam Phys.

[9] National Institute for Health and Care Excellence (N.I.C.E.). Eating disorders: recognition and treatment. 2020. 33689256

[10] Academy for Eating Disorders (AED). Eating disorders: a guide to medical care. 2021.

[11] Johnson LB, Boyd LD, Rainchuso L, Rothman A, Mayer B (2017). Eating disorder professionals’ perceptions of oral health knowledge. Int J Dent Hyg.

[12] Flynn M, Austin A, Lang K, Allen K, Bassi R, Brady G (2021). Assessing the impact of first episode rapid early intervention for eating disorders on duration of untreated eating disorder: a multi-centre quasi-experimental study. Eur Eat Disord Rev.

[13] Austin A, Flynn M, Richards K, Hodsoll J, Duarte TA, Robinson P (2021). Duration of untreated eating disorder and relationship to outcomes: a systematic review of the literature. Eur Eat Disord Rev.

[14] Mahr F, Farahmand P, Bixler EO, Domen RE, Moser EM, Nadeem T (2015). A national survey of eating disorder training. Int J Eat Disord.

[15] Girz L, Robinson AL, Tessier C (2014). Is the next generation of physicians adequately prepared to diagnose and treat eating disorders in children and adolescents?. Eat Disord.

[16] Ayton A, Ibrahim A (2018). Does UK medical education provide doctors with sufficient skills and knowledge to manage patients with eating disorders safely?. Postgrad Med J.

[17] Peters MD, Godfrey CM, Khalil H, McInerney P, Parker D, Soares CB (2015). Guidance for conducting systematic scoping reviews. Int J Evid Based Healthc.

[18] Tricco AC, Lillie E, Zarin W, O'Brien KK, Colquhoun H, Levac D (2018). PRISMA extension for scoping reviews (PRISMA-ScR): checklist and explanation. Ann Intern Med.

[19] Burgard M, Canevello A, Mitchell J, De Zwaan M, Crosby R, Wonderlich S (2003). Dental practitioners and eating disorders. Eat Disord.

[20] DeBate RD, Bleck JR, Raven J, Severson H (2017). Using intervention mapping to develop an oral health e-curriculum for secondary prevention of eating disorders. J Dent Educ.

[21] DeBate RD, Cragun D, Gallentine AA, Severson HH, Shaw T, Cantwell C (2012). Evaluate, assess, treat: development and evaluation of the EAT framework to increase effective communication regarding sensitive oral-systemic health issues. Eur J Dent Educ.

[22] DeBate RD, Plichta SB, Tedesco LA, Kerschbaum WE (2006). Integration of oral health care and mental health services: dental hygienists’ readiness and capacity for secondary prevention of eating disorders. J Behav Health Serv Res.

[23] Debate RD, Severson H, Zwald ML, Shaw T, Christiansen S, Koerber A (2009). Development and evaluation of a web-based training program for oral health care providers on secondary prevention of eating disorders. J Dent Educ.

[24] DeBate RD, Severson HH, Cragun D, Bleck J, Gau J, Merrell L (2014). Randomized trial of two e-learning programs for oral health students on secondary prevention of eating disorders. J Dent Educ.

[25] DeBate RD, Shuman D, Tedesco LA (2007). Eating disorders in the oral health curriculum. J Dent Educ.

[26] Debate RD, Tedesco LA (2006). Increasing dentists’ capacity for secondary prevention of eating disorders: identification of training, network, and professional contingencies. J Dent Educ.

[27] DeBate RD, Tedesco LA, Kerschbaum WE (2005). Knowledge of oral and physical manifestations of anorexia and bulimia nervosa among dentists and dental hygienists. J Dent Educ.

[28] Debate RD, Vogel E, Tedesco LA, Neff JA (2006). Sex differences among dentists regarding eating disorders and secondary prevention practices. J Am Dent Assoc.

[29] DiGioacchino RF, Keenan MF, Sargent R (2000). Assessment of dental practitioners in the secondary and tertiary prevention of eating disorders. Eat Behav.

[30] Frimenko KM, Murdoch-Kinch CA, Inglehart MR (2017). Educating dental students about eating disorders: perceptions and practice of interprofessional care. J Dent Educ.

[31] Galindo DF, Butura CC (2014). Immediate loading of dental implants in the esthetic region using computer-guided implant treatment software and stereolithographic models for a patient with eating disorders. J Prosthodont.

[32] Giraudeau N, Camman P, Pourreyron L, Inquimbert C, Lefebvre P (2021). The contribution of teledentistry in detecting tooth erosion in patients with eating disorders. Digit Health.

[33] Johansson AK, Johansson A, Nohlert E, Norring C, Astrom AN, Tegelberg A (2015). Eating disorders: knowledge, attitudes, management and clinical experience of Norwegian dentists. BMC Oral Health.

[34] Lee JY, Kim SW, Kim JM, Shin IS, Yoon JS (2015). Two cases of eating disorders in adolescents with dental braces fitted prior to the onset of anorexia nervosa. Psychiatry Investig.

[35] Turhani D, Ohlmeier KH, Sutter W, Kielbassa AM (2019). Undesirable course of an oral implant rehabilitation in a patient with a long history of bulimia nervosa: case report and review of the literature. Quintessence Int.

[36] Back-Brito GN, da Mota AJ, de Souza Bernardes LA, Takamune SS, Prado Ede F, Cordas TA (2012). Effects of eating disorders on oral fungal diversity. Oral Surg Oral Med Oral Pathol Oral Radiol.

[37] Boillot A, Ringuenet D, Kapila Y, Pallier A, Colon P, Bouchard P (2020). High serum ferritin levels are associated with a reduced periodontium in women with anorexia nervosa. Eat Weight Disord.

[38] de Moor RJ (2004). Eating disorder-induced dental complications: a case report. J Oral Rehabil.

[39] Dynesen AW, Bardow A, Petersson B, Nielsen LR, Nauntofte B (2008). Salivary changes and dental erosion in bulimia nervosa. Oral Surg Oral Med Oral Pathol Oral Radiol Endod.

[40] Emodi-Perlman A, Yoffe T, Rosenberg N, Eli I, Alter Z, Winocur E (2008). Prevalence of psychologic, dental, and temporomandibular signs and symptoms among chronic eating disorders patients: a comparative control study. J Orofac Pain.

[41] Hermont AP, Pordeus IA, Paiva SM, Abreu MH, Auad SM (2013). Eating disorder risk behavior and dental implications among adolescents. Int J Eat Disord.

[42] Imai T, Michizawa M (2013). Necrotizing sialometaplasia in a patient with an eating disorder: palatal ulcer accompanied by dental erosion due to binge-purging. J Oral Maxillofac Surg.

[43] Johansson AK, Norring C, Unell L, Johansson A (2012). Eating disorders and oral health: a matched case-control study. Eur J Oral Sci.

[44] Lifante-Oliva C, Lopez-Jornet P, Camacho-Alonso F, Esteve-Salinas J (2008). Study of oral changes in patients with eating disorders. Int J Dent Hyg.

[45] Lourenco M, Azevedo A, Brandao I, Gomes PS (2018). Orofacial manifestations in outpatients with anorexia nervosa and bulimia nervosa focusing on the vomiting behavior. Clin Oral Investig.

[46] Lundgren JD, Williams KB, Heitmann BL (2010). Nocturnal eating predicts tooth loss among adults: results from the Danish MONICA study. Eat Behav.

[47] Otsu M, Hamura A, Ishikawa Y, Karibe H, Ichijyo T, Yoshinaga Y (2014). Factors affecting the dental erosion severity of patients with eating disorders. Biopsychosoc Med.

[48] Pallier A, Karimova A, Boillot A, Colon P, Ringuenet D, Bouchard P (2019). Dental and periodontal health in adults with eating disorders: a case-control study. J Dent.

[49] Paszynska E, Hernik A, Slopien A, Roszak M, Jowik K, Dmitrzak-Weglarz M (2022). Risk of dental caries and erosive tooth wear in 117 children and adolescents’ anorexia nervosa population-a case-control study. Front Psychiatry.

[50] Paszynska E, Schlueter N, Slopien A, Dmitrzak-Weglarz M, Dyszkiewicz-Konwinska M, Hannig C (2015). Salivary enzyme activity in anorexic persons-a controlled clinical trial. Clin Oral Investig.

[51] Range H, Pallier A, Ali A, Huas C, Colon P, Godart N (2021). Risk factors for oral health in anorexia nervosa: comparison of a self-report questionnaire and a face-to-face interview. Int J Environ Res Public Health.

[52] Sales-Peres SHC, Araujo JJ, Marsicano JA, Santos JE, Bastos JRM (2014). Prevalence, severity and etiology of dental wear in patients with eating disorders. Eur J Dent.

[53] Shaughnessy BF, Feldman HA, Cleveland R, Sonis A, Brown JN, Gordon CM (2008). Oral health and bone density in adolescents and young women with anorexia nervosa. J Clin Pediatr Dent.

[54] Ximenes R, Couto G, Sougey E (2010). Eating disorders in adolescents and their repercussions in oral health. Int J Eat Disord.

[55] Strużycka I, Lussi A, Boguslawska-Kapala A, Rusyan E. Prevalence of erosive lesions with respect to risk factors in a young adult population in Poland-across-sectional study. Clin Oral Investig. 2017;21(7):2197-203. 10.1007/s00784-016-2012-z PMC5559558 27981411

[56] Conviser JH, Fisher SD, Mitchell KB (2014). Oral care behavior after purging in a sample of women with bulimia nervosa. J Am Dent Assoc.

[57] Dynesen AW, Gehrt CA, Klinker SE, Christensen LB (2018). Eating disorders: experiences of and attitudes toward oral health and oral health behavior. Eur J Oral Sci.

[58] Johansson AK, Mjanger Ovretvedt T, Reinholtsen KK, Johansson A (2022). Eating disorders: an analysis of self-induced vomiting, binge eating, and oral hygiene behavior. Int J Clin Pract.

[59] Johansson AK, Norring C, Unell L, Johansson A (2020). Diet and behavioral habits related to oral health in eating disorder patients: a matched case-control study. J Eat Disord.

[60] Sharifian MJ, Pohjola V, Kunttu K, Virtanen JI (2021). Association between dental fear and eating disorders and body mass index among finnish university students: a national survey. BMC Oral Health.

[61] Sirin Y, Yucel B, Firat D, Husseinova-Sen S (2011). Assessment of dental fear and anxiety levels in eating disorder patients undergoing minor oral surgery. J Oral Maxillofac Surg.

[62] Willumsen T, Graugaard PK (2005). Dental fear, regularity of dental attendance and subjective evaluation of dental erosion in women with eating disorders. Eur J Oral Sci.

[63] Anderson K, Accurso EC, Kinasz KR, Le Grange D (2017). Residents’ and fellows’ knowledge and attitudes about eating disorders at an academic medical center. Acad Psychiatry.

[64] Frydrych AM, Davies GR, McDermott BM (2005). Eating disorders and oral health: a review of the literature. Aust Dent J.

[65] Lo Russo L, Campisi G, Di Fede O, Di Liberto C, Panzarella V, Lo ML (2008). Oral manifestations of eating disorders: a critical review. Oral Dis.

[66] Kisely S, Baghaie H, Lalloo R, Johnson NW (2015). Association between poor oral health and eating disorders: systematic review and meta-analysis. Br J Psychiatry.

[67] Monda M, Costacurta M, Maffei L, Docimo R (2021). Oral manifestations of eating disorders in adolescent patients. A review Eur J Paediatr Dent.

